# Rapid and Versatile Biosensing of Liposome Encapsulation Efficiency Using Electrical Conductivity Sensor

**DOI:** 10.3390/bios13090878

**Published:** 2023-09-08

**Authors:** Tatiane Melo Pereira, Cínthia Caetano Bonatto, Luciano Paulino Silva

**Affiliations:** 1 Embrapa Recursos Genéticos e Biotecnologia, Laboratório de Nanobiotecnologia (LNANO), Parque Estação Biológica, Final W5 Norte, Brasília 70770-917, DF, Brazil; tatimepr@gmail.com (T.M.P.); cinthiabonatto@gmail.com (C.C.B.); 2 Postgraduate Program in Life Sciences (Molecular Biology), University of Brasilia (UnB), Brasília 70910-900, DF, Brazil; 3 Postgraduate Program in Pharmaceutical Sciences, Federal University of Paraná, Botanical Garden, Curitiba 80210-170, PR, Brazil

**Keywords:** extract-containing liposomes, electrical conductivity, glassy platinum electrode, secondary metabolites

## Abstract

Liposomes are prominent nanosystems for drug delivery, with potential extending beyond isolated drugs. Ethanol-aqueous plant extracts can be encapsulated within liposomes to protect bioactive compounds (secondary metabolites) from rapid oxidation and enable sustained release. Determining which compound classes are present in each extract and the encapsulation efficiency (EE) of these extracts in liposomes is crucial for nanocarrier functionality. This involves assessing the ratio of bioactive substances within liposomes to the total content. However, quantifying EE for non-isolated compounds poses challenges due to the need for advanced analytical equipment and biosensing approaches. This study introduces an innovative method for EE quantification, using a conductivity electrode (k = 0.842/cm) to establish an EE biosensing technology. By correlating dynamic light scattering (DLS), zeta potential (ZP), and electrical conductivity (Cnd) data with the conductivity meter’s calibration curve, a robust relationship between the free extract concentration and Cnd (r^2^ ≥ 0.950) was established. Lavender-loaded liposomes demonstrated an EE of 56.33%, while wormwood and oregano formulations exhibited high EEs of 94.33% and 91.70%, respectively. In contrast, sage-loaded liposomes exhibited an inadequate EE, encapsulating only approximately 0.57% of the extract. The straightforward quantification of the free extract within liposome formulations, compared to more complex approaches, could facilitate EE determination and support future characterizations.

## 1. Introduction

Liposomes (Lp) are concentric vesicles that harbor hydrophilic cores and hydrophobic bilayers. This configuration makes them suitable for carrying compounds and/or bioactives with similar affinity [1]. Liposomes have diverse applications in nanomedicine, food technology, and even agriculture [2,3,4,5]. This carrier confers increased stability to the transported compound while facilitating sustained release [6].

Extracts derived from plants contain a variety of compounds, some of which exhibit antimicrobial activities [7,8] or are used as herbal medicines [9]. Among the well-recognized secondary metabolites, flavonoids are valued as dietary supplements and exhibit antimicrobial properties [10]. Tannins, which have traditionally been used in leather tanning, also have antitumor and antioncogenic activities [11]. Saponins have undergone scrutiny for their anticholesterol and anticancer potentials [12], while anthraquinones, which were once used as dyes, also serve as laxatives, antimicrobial agents, and anti-inflammatory remedies [13]. However, plant extracts rich in these metabolites are susceptible to rapid oxidation [14], highlighting the importance of encapsulation.

The EE is an extremely relevant characterization step, as it allows one to determine the amount of substance that was effectively encapsulated within the liposomes. This is crucial to understand whether the formulation is actually fulfilling its purpose of delivering and controlling the release of the substance. However, the analytical techniques that are currently available for calculating the EE when liposomes carry complex substances such as plant extracts are complex and expensive, such as chromatography [15]. Commonly, chromatography requires the separation and identification of individual compounds. Other techniques can also be used to calculate EE, but overall they are not simple, rapid, or versatile [16,17].

The current study presents an innovative biosensing approach that uses an electrical conductivity electrode to evaluate the EE of liposomes carrying complex mixtures, such as plant extracts, without the need for compound isolation. The novelty of this approach lies in the development of a simple and inexpensive method for indirectly quantifying EE in plant-extract-loaded liposomes. This method utilizes an electrical conductivity sensor to measure the total amount of solids dissolved in a sample. By correlating the electrical conductivity value with the concentration of a plant extract sample, we can indirectly determine the EE without the need for time-consuming and expensive analytical techniques. This method is much simpler and faster than chromatography.

This proposed method benefits from the fact that electrical conductivity measures the entirety of solids dissolved within a sample [18]. Recent research underlines the myriad factors that can influence electrical conductivity, including material properties, as well as the configuration and dimensions of dissolved solids [19]. Thus, for the proposed biosensor to fulfill its function, it is sufficient that the free extracts, the liposomes that are carrying them, and the empty liposomes undergo an ultrafiltration process. The conductivity electrode is then submerged in the ultrafiltered content, which provides a reading that can be correlated to the extract concentration. Understanding the direct correlation between extract concentration and electrical conductivity will lay the foundation for assessing EE, thereby presenting an underexplored avenue in the realm of nanobiotechnology applications.

## 2. Materials and Methods

### 2.1. Materials

For the preparation of solutions, ultrapure water (type I water) was utilized alongside analytical-grade reagents, including sodium hydroxide (Neon, Suzano, SP, Brazil); ammonium hydroxide (Vetec, Duque de Caxias, RJ, Brazil); gelatin powder (Dinâmica, Indaiatuba, SP, Brazil); copper acetate (Êxodo, Sumaré, SP, Brazil); hydrochloric acid (Vetec, Duque de Caxias, RJ, Brazil); and sulfuric acid (Vetec, Duque de Caxias, RJ, Brazil) for the preparation of some solutions. Alcoholic solutions were prepared using ethanol (Dinâmica, Indaiatuba, SP, Brazil) combined with aluminum chloride (Dinâmica, Indaiatuba, SP, Brazil); ferric chloride (Proquímios, Rio de Janeiro, RJ, Brazil); quinine hydrochloride (Dinâmica, Indaiatuba, SP, Brazil); and vanillin (Dinâmica, Indaiatuba, SP, Brazil). Solvents such as hexane (Sigma-Aldrich, Darmstadt, Hesse, Germany); methyl alcohol (Vetec, Duque de Caxias, RJ, Brazil); and acetone (CRQ Química, Diadema, SP, Brazil) were also employed. Phytochemical screening utilized metallic magnesium shavings (Dinâmica, Indaiatuba, SP, Brazil); dried botanical material lavender and wormwood (Produtos Melvina, Goiânia, GO, Brazil); and oregano and sage (Produtos Gizele, Brasília, DF, Brazil), along with commercially procured bar soap.

### 2.2. Production of Ethanolic Extracts

Ethanolic/aqueous extracts were derived from commercially obtained dry botanical material encompassing lavender (*Lavandula officinalis*), wormwood (*Artemisia absinthium*), oregano (*Origanum vulgare*), and sage (*Salvia officinalis*). A maceration process ensued, involving 5 g of dry botanical material immersed in 100 mL of 70% ethanol for 24 h at 4 °C. Subsequent filtration and concentration were performed using a rotary evaporator with a heating bath set at 40 °C and a cooling bath set at 10 °C under a pressure of 400 Pa. Freeze-drying yielded a powdered extract, which was then resuspended in ultrapure water to achieve a final concentration of 2.5 g/mL.

### 2.3. Phytochemical Screening

Ethanolic/aqueous extracts underwent a battery of reactions to demonstrate the presence of secondary metabolites, as detailed in Table 1. The characterization involved flavonoids, anthraquinones, tannins, and saponins. Standard compounds were employed as positive controls for validation.

Flavonoids: In the Shinoda reaction, two metallic magnesium fragments (2 × 2 mm) were introduced into 2 mL extracts, followed by the addition of 1 mL concentrated hydrochloric acid. The emergence of a reddish color indicated the presence of flavones or flavonols. For the aluminum chloride reaction, a drop of the extract was applied to filter paper at various points. Subsequently, a drop of 1% alcoholic solution of aluminum chloride was added to one of the spots, and the reaction was observed under ultraviolet light. The positive reaction was discerned through heightened fluorescence. The ferric chloride reaction involved the exposure of 2 mL extracts to 4 drops of a 25% alcoholic solution of ferric chloride. A color shift to blue, green, or brown indicated a positive reaction. Lastly, for the anthocyanin characterization reaction, 10 mL of the extracts underwent alkalization with 5% sodium hydroxide until reaching pH 10.0. Subsequent acidification with 10% hydrochloric acid until pH 3.0 induced a color change—red for acidic and blue for basic—indicating the presence of anthocyanins. To authenticate the presence of flavonoids, chamomile (*Matricaria chamomilla*) was employed as a positive control.

Anthraquinones: In the direct Borntrager reaction, 5 mL of the extract mixed with 3 mL of hexane underwent organic phase extraction, followed by the addition of 2 mL of a 10% ammonium hydroxide solution. In the indirect Borntrager reaction, 5 mL of the extracts were subjected to 1 mL 10% hydrochloric acid, boiled for 3 min, and then extracted with 1 mL hexane. Subsequently, a 10% ammonium hydroxide solution was added to the aqueous phase. The ferric chloride precipitation reaction employed the same acid solution obtained in the indirect Borntrager reaction, supplemented with 2 mL of 10% ferric chloride. After boiling for 3 min and extraction with 2 mL of hexane, the organic phase underwent removal. Then, 2 mL of 10% ammonium hydroxide solution was added. In all anthocyanin reactions, the development of a red or violet color in the aqueous phase signified the presence of free anthraquinones in the plant drug. For anthraquinone presence verification, Cascara sagrada *(Rhamnus purshiana* cortex) served as a positive control, while water was used as a negative control.

Tannins: For the ferric chloride reaction, 3 drops of a 2% alcoholic solution of ferric chloride were added and stirred well in 2 mL of extract, resulting in a greenish-blue or gray color indicative of phenolic compounds. The protein reaction involved adding 2 drops of 10% hydrochloric acid and 5 drops of 2.5% gelatin solution to 2 mL of the extract, leading to the appearance of a precipitate indicating the presence of tannins. In the alkaloid precipitation reaction, 4 drops of 8% quinine alcoholic solution were added to 2 mL of extract, and turbidity or precipitate formation indicated tannin presence. For the copper acetate reaction, 3 drops of 3% copper acetate solution were added to 2 mL of extract, with turbidity or precipitate formation signifying tannin presence. Finally, the condensed tannin reaction involved adding 0.5 mL of 1% alcoholic vanillin solution and 1 mL of concentrated hydrochloric acid to 2 mL of extract, resulting in a red color development, indicative of condensed tannins. To characterize tannins in the sample, the aqueous extract of Espinheira Santa (*Maytenus ilicifolia*) was used as a positive control.

Saponis: The first test, named “characterization of foam formation” entailed the vigorous shaking of 2 mL of the extract and a comparison with a tube containing water and ordinary bar soap. The second test involved adding 5 drops of 20% sulfuric acid 10 min after the first test. The presence of persistent foam upon acid addition signaled the existence of saponins in the drug. For saponin characterization, the standard drug Salsa-parrilha (*Smilax* ssp.) served as a positive control.

### 2.4. Liposome Production

Botanical material (leaves and petioles) was collected from the Botanical Garden of Brasília (JBB) with the proper authorization from the JBB (007/2019), from the Biodiversity Authorization and Information System—SISBIO, under permit number 74283-1 and from the National System of Genetic Heritage Management and Associated Traditional Knowledge—SISGEN under license number A078201. Collected plants from the same species, which were also obtained commercially, were duly identified. 

To extract the phospholipids from the botanical material, 0.250 g of the leaves were immersed in 25 mL of acetone for 24 h to remove the chlorophyll. After this period, the leaves were macerated, and the extraction was performed with solvents: water, chloroform, and methanol, in the proportion of 1:1.4:2.6, adapted from the patent WO2016119030A1. The organic portion, containing phospholipids, was dried using a rotary evaporator with an immersion bath at 40 °C and ultra-thermostatic cooling at 10 °C for 1 h. The resulting lipid film from each species underwent a hydration process with ultrapure water (empty liposomes) and ethanol-aqueous extract at a concentration of 40 mg/mL (full liposomes). This production yielded a total of 8 liposomal nanosystems, since for each one of four plant species, two types of liposomes were formulated: empty liposomes (phospholipids of a plant resuspended with water) and full liposomes (phospholipids of a plant resuspended with extract of the same plant).

### 2.5. Characterization of Liposomes

To assess the hydrodynamic diameter (HD), polydispersion index (PdI), zeta potential (ZP), and electrical conductivity (Cnd), samples were diluted 1:10 in ultrapure water and evaluated using a DTS 1070 cuvette and 0.950 mL of the sample in a ZetaSizer Nano ZS (Malvern, UK). The HD and PdI were evaluated by dynamic light scattering (DLS), and ZP and Cnd were evaluated by electrophoretic mobility (Cnd-EM). DLS analysis was performed at an angle of 173° using a He-Ne laser (4 mW) operating at 633 nm. DLS measurements were conducted in automatic mode, while ZP and Cnd data were acquired in manual mode with 20 readings for each replicate. Three measurements of each sample were obtained at 25 °C.

### 2.6. Analysis of Electrical Conductivity

Electrical conductivity (Cnd) measurements of the samples were conducted using a microprocessed conductivity meter with a conductivity electrode possessing a vitreous body (k = 0.842/cm) and a resolution of 0.1 μS (Quimis—Q795M2). The electrode consisted of black platinum plates with a constant of 1, along with a temperature sensor T-818-B-6. The evaluations were carried out using an electrode with electronic precision for conductivity, providing ±0.5% full-scale (FS) accuracy, a stability of ±0.3% FS, and a reproducibility of ±0.16% FS. The electrode evaluated the conductivity based on the total dissolved solids. Before each measurement, the conductivity electrode was immersed in 1 mL of ultrapure water, and the reading was recorded. Then, it was thoroughly dried. Between each conductivity reading, the electrode was rinsed abundantly with distilled water and properly dried.

To establish a calibration curve, the extract was filtered using an Amicon^®^ 3 kDa ultrafilter at 5 °C. The process involved sequential centrifugation steps: 268 g for 30 min, followed by 1073 g for 30 min, and finally 2415 g for 60 min. The material that passed through the filter underwent serial dilution starting from the 1:2 dilution condition applied in the formation of liposomes (40 mg/mL) up to a dilution of 1:128, using a dilution factor of 2.

To assess precision and repeatability and calculate the standard deviation, three independent readings of each sample were taken. This process allowed the construction of a calibration curve for each sample based on a graph with logarithmized axes, ensuring equidistant evaluation points. All measurements were performed using 1.5 mL of the sample in plastic cuvettes at room temperature (22 °C).

### 2.7. Encapsulation Quantification

To comprehend the encapsulation rate, both empty and full liposomes underwent ultrafiltration using an Amicon^®^ ultrafilter with a 3 kDa cutoff at 5 °C. The process involved sequential centrifugation steps: 268 g for 30 min, followed by 1073 g for 30 min, and finally 2415 g for 60 min. Subsequently, the filtered material was collected and diluted to 1:5, and the conductivity reading was taken on a Quimis conductivity meter, always at a temperature of 22 °C. The quantification of encapsulation was performed by correlating the obtained reading, adjusted for the values obtained from the conductivity of ultrapure water and the filtrate of empty liposomes, both multiplied by the dilution factor. This value was then applied to the equation of the line obtained in the previous step.

### 2.8. Statistical Analysis

Mean and standard deviation (SD) calculations for the datasets were performed using Microsoft Office Excel software version 2307 Build 16.0.16626.20170 (Home and Business 2016). The size distribution curves, calibration, and linear regression curves, along with the equation of the line, were obtained using SciDAVis 2.7 software. Statistical analysis, specifically one-way ANOVA, was conducted using PAST version 2.17b.

## 3. Results

### 3.1. Phytochemical Screening

Regarding the analysis of the flavonoid phytochemical group, the Shinoda reaction yielded positive results for all samples (lavender, wormwood, oregano, and sage). A more intense reddish color developed in the sage and lavender samples, suggesting the presence of flavones or flavonols in the plant material. In the case of the aluminum chloride reaction, only the oregano sample exhibited enhanced fluorescence under UV light. In the ferric chloride precipitation reaction, all samples displayed a blue-green color, indicating the presence of phenolic compounds, with oregano exhibiting a more intense hue. Concerning anthocyanin characterization, positive reactions were observed only in oregano and lavender, with their color changing according to the pH, signifying the presence of anthocyanins in the plant material. To confirm the presence or absence of a phytochemical group, a minimum of 80% positive reactions is required. Consequently, only lavender and oregano confirmed the presence of the flavonoid phytochemical group.

In the evaluation of the anthraquinone metabolite using the direct Borntrager reaction, oregano displayed a faint reddish color in the aqueous phase. Conversely, all other samples (lavender, wormwood, and sage) exhibited negative reactions, implying no color change. Hence, the presence of anthraquinones could not be inferred in any of the extracts used.

For the assessment of tannins, all samples (lavender, wormwood, oregano, and sage) displayed a grayish-blue color in the ferric chloride reaction, suggesting the presence of phenolic compounds in the plant material. In the protein reaction, only lavender and oregano demonstrated turbidity, indicating the presence of tannins. The alkaloid precipitation reaction resulted in precipitate formation for all samples, indicating the presence of tannins in the solution. Similarly, the copper acetate reaction showed turbidity and/or precipitation for all samples, once again pointing to the presence of tannins. Lastly, in the condensed tannin reaction, only lavender indicated the development of a reddish color, indicating the presence of condensed tannins. In the assessment of tannins, only lavender and oregano exhibited positive reactions in at least 80% of the tests.

Initial vigorous stirring produced foam in all samples; however, persistent foam formation upon acid addition was observed only in lavender, wormwood, and sage, indicating the presence of saponins. A summary of all results can be found in Table 2.

### 3.2. Characterization of Liposomes

**Figure 1:**
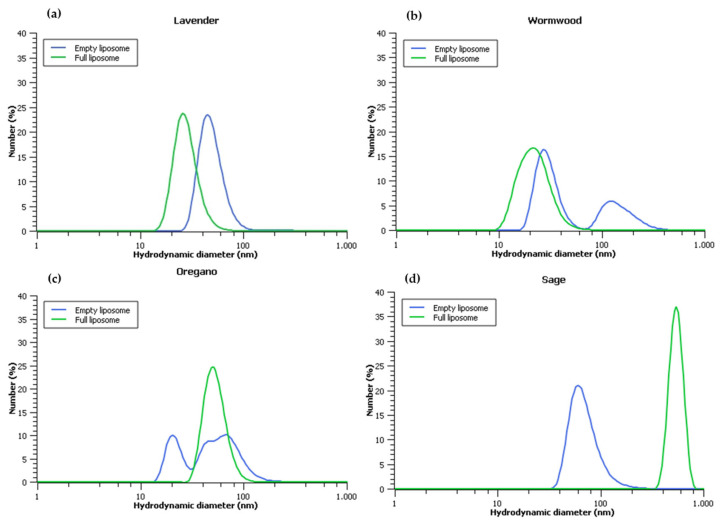
Size distribution (hydrodynamic diameter) as number of liposomes produced using phospholipids extracted from various plants: (**a**) lavender, (**b**) wormwood, (**c**) oregano, and (**d**) sage. Liposomes were loaded with either ultrapure water (blue line, empty liposome) or ethanolic/aqueous phyto-extract from the same plant as the source of the phospholipids (green line, full liposome).

The HD of the liposomes was evaluated to assess their size and uniformity. The HD is a measure of the size of a particle suspended in a fluid, and it is important to evaluate the HD of liposomes because it can affect their stability and biological activity. All eight nanosystems exhibited the formation of nanostructures, as observed through DLS analysis (Figure 1). Empty liposomes were evaluated to determine the baseline HD of the liposomes without any added constituents. Full liposomes were evaluated to determine the effect of loading the liposomes with plant extract on their HD. The results showed that the HDs of the full liposomes were overall different from those of empty liposomes, but there was no clear consensus regarding whether the size increased or decreased. This suggested that the plant extract may interact with the phospholipids in the liposomes differently depending on the plant species used. Specifically, in the case of sage, a notable increase in HD was observed when the liposome was loaded with plant extract, as depicted in Figure 1. Studies have shown that the content of a liposome may not have a significant impact on its HD, but lipid composition has a strong influence on this characteristic [20], as observed in the present study. It is noteworthy that DLS analysis to determine HD can be affected by the shape and aggregation state of the liposomes. When individual extracts were subjected to DLS analysis, none of them showed evidence of nanostructures. Instead, they revealed micrometric agglomerates exceeding 1000 nm in size. In the case of oregano, the reading process was terminated by the device, suggesting the absence of nano- or microstructures.

As depicted in Table 3, the PdI value for the lavender sample indicated statistical equivalence between the extract and the full liposome, with recorded values of 0.385 ± 0.004 and 0.407 ± 0.044, respectively. Both exhibited lower polydispersity than the empty liposome, which registered a PdI of 0.604 ± 0.017. Evaluating the ZP of wormwood, a notable and statistically significant increase in the modulus value was apparent in the full liposome (−34.4 ± 3.3 mV) in comparison to the extract (−14.0 ± 0.6 mV) and empty liposome (−14.4 ± 0.6 mV). Moreover, Cnd-EM showcased higher values for both the extract and the full liposome, in contrast to the empty liposome alone.

Within the wormwood species, no statistically significant variance was observed in PdI across the samples. However, ZP analysis demonstrated an increased modulus value with the formation of full liposomes. Notably, Cnd-EM displayed statistically significant differences across all samples, with the most pronounced increase occurring upon the formulation of full liposomes.

In the case of oregano, PdI assessment was unsuccessful for the extract due to equipment termination, strongly suggesting the absence of nanostructures. It is noteworthy that the full liposome displayed a statistically significant reduction in PdI (0.488 ± 0.056) compared to the empty liposome (0.686 ± 0.104). Analyzing the ZP of oregano, it was evident that liposome formation led to a substantial increase in the modulus value compared to the extract. Additionally, the electrical conductivity also notably increased with the formulation of the full liposome.

In the sage sample, PdI exhibited distinctions among the three samples, with the lowest value noted in the empty liposome (0.318 ± 0.034) and the highest in the full liposome (0.984 ± 0.028). The ZP remained statistically consistent between the extract and the filled liposome, with only the empty liposome displaying a distinct and smaller modulus value (−11.5 ± 1.5 mV). Similarly, Cnd-EM demonstrated statistical similarity between the extract and the filled liposome, with deviations observed solely in the empty liposome.

### 3.3. Standalone Electrical Conductivity Analysis

In addition to the Cnd-EM performed using the ZetaSizer equipment, further biosensing measurements were performed using standalone conductivimeter equipment. To construct the calibration curves, the Cnd of the ethanolic/aqueous extracts was measured at different concentrations (serial dilutions). The electrical conductivity of ultrapure water was also measured, resulting in values of 2.17/2.09/2.06 µs/cm. The water conductivity value was subtracted from the extract ultrafiltrate readings of each species. The results showed that there was a linear relationship between the Cnd and the concentration of the extracts.

**Figure 2:**
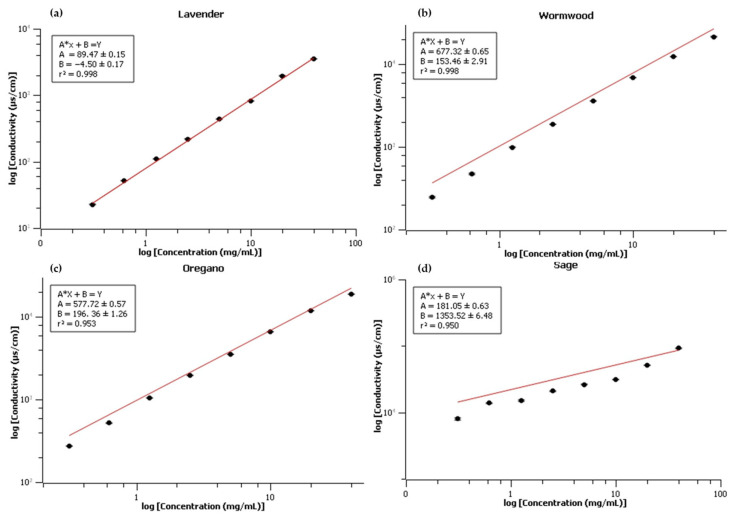
Calibration curve of ethanolic/aqueous extract concentration (mg/mL) and corresponding Cnd (µs/cm) for (**a**) lavender, (**b**) wormwood, (**c**) oregano, and (**d**) sage. The red line demonstrates the linearity of the data, along with the equation indicated in each graph.

Figure 2 illustrates the correlation and linearity between the dilution of the plant extracts and the Cnd, with a correlation coefficient greater than 0.95 observed for all species. Several studies have established a correlation between electrical conductivity and the concentration of salts and organic matter in solution [18,21]. The intra-assay variation in Cnd readings (the difference between the highest and the lowest readings at the same point) was ≤1.5% for the lavender species, ≤3.4 % for the wormwood species, ≤3.4% for the oregano species, and ≤3.1% for the sage species. These results indicate that the method provided satisfactory precision and low variance in correlating Cnd with the concentration of the extract used.

Finally, it has previously been demonstrated that calibration curves with an r^2^ ≥ 0.95 are a good standard to determine the linearity of a curve [22]. With such linearity, it became possible to determine the equation of the line for each species, as follows:

Lavender:

89.47x−4.5=y

Wormwood:

677.32x+153.46=y

Oregano:

577.72x+196.36=y

Sage:

181.05x+1353.52=y

### 3.4. Quantification of Encapsulation

The quantification of EE, a key aspect in the utilization of engineered nanomaterials, was achieved using an ingenious biosensing approach. To calculate the extract concentration that was not encapsulated in the liposomes, an ultrafiltration process was performed, and the ultrafiltrate was measured by a Cnd electrode. The reading obtained here was perfectly integrated into the formula explained in the preceding section. With the initial concentration set at 40 mg/mL, this fundamental information was the foundation for complex calculations.

Based on this foundation, the concentration of plant extract that resided outside the liposomal confines was precisely determined. This was achieved by aligning the calibration curve for each extract with the Cnd values of the ultrafiltrated liposomes, shown in Table 4. The “*y*” value of the formula in the previous section was then added for each species. Finally, the percentage of extract that remained uncontained within the liposomes (free) was determined, revealing an incisive biosensing-driven quantification strategy. This approach enabled the indirect quantification of the total entrapment of each extract within liposomes.

In the case of lavender, the incisive analysis revealed the presence of 17.47 ± 0.15 mg/mL of extract equivalent outside the liposomes, constituting 43.67 ± 0.37% of the initial concentration. For wormwood, a corresponding analysis indicated 2.27 ± 0.05 mg/mL of the extract existing outside the liposomes, equating to 5.67 ± 0.12% of the initial concentration. As for oregano, 3.32 ± 0.07 mg/mL of the extract remained outside, representing 8.30 ± 0.17% of the initial concentration. Lastly, sage exhibited 39.77 ± 0.77 mg/mL of extract remaining outside, encompassing a staggering 99.43 ± 1.93% of the initial concentration. These perceptive findings underscore the variegated encapsulation dynamics inherent in different extracts. This was to be expected, because when the phospholipid content for liposome formation varied, there was typically a variation in EE, as observed by other authors [17]. In the present study, the sources and consequently the phospholipid content were modulated. While extracts like wormwood and oregano experienced pronounced encapsulation, sage exhibited a markedly divergent behavior, with a substantial majority of its bioactive constituents remaining beyond the liposomal boundaries.

In summary, the analyses revealed an EE of 56.33 ± 0.37%, 94.33 ± 0.12%, 91.70 ± 0.17%, and 0.57 ± 1.93% for full Lp containing lavender, wormwood, oregano, and sage extracts, respectively. These results indicated that the encapsulation of the extracts within liposomes was not consistent across all samples. Notably, for certain extracts like wormwood and oregano, a significant proportion of the active compounds was successfully encapsulated, while for others like sage, the encapsulation was insufficient.

## 4. Discussion

Liposomes, serving as proficient carriers for bioactive compounds like plant extracts, offer a multitude of advantages, encompassing safeguarding fragile substances, extending circulation, and achieving targeted tissue delivery [23]. However, a precise and swift assessment of bioactive compounds’ EE within liposomes is crucial for ensuring the efficacy and quality of these carriers [24,25].

EE assumes a pivotal role in scrutinizing the successful incorporation of molecules into liposomes, particularly vital for nanosystems entrusted with molecular cargo. This relevance emerges from the susceptibility of molecules left exterior to liposomes, rendering them vulnerable to oxidation and swift degradation [26]. Moreover, effective drug encapsulation has the potential to augment oral bioavailability [27].

Traditional approaches to characterizing EE often entail time-intensive techniques, such as chromatography, which not only curtail research efficiency but also introduce variability due to intricate manual handling and processing steps, particularly when considering plant extracts. Hence, the quest for expeditious, precise, and adaptable methodologies for liposome encapsulation evaluation remains a realm of investigation [28]. A notable technique utilized for EE evaluation involves fluorimetric measurement [29], contingent upon the active ingredient possessing a distinctive absorption peak. Nonetheless, in scenarios involving intricate solutions like plant extracts, the absence of a discernible peak can render EE characterization challenging.

Plant secondary metabolites encompass a diverse range of chemical compounds produced as part of plants’ secondary metabolism, often non-essential for primary growth yet pivotal in interacting with the environment. Within this spectrum, alkaloids, flavonoids, and tannins play multifaceted roles, spanning applications in medicine, nutrition, and industry [30]. The phytochemical evaluation of the extracts revealed the presence of a varied array of compound classes: flavonoids, tannins, and saponins in lavender; saponins in wormwood; flavonoids and tannins in oregano; and saponins in sage. In fact, a prior phytochemical screening of the ethanolic/aqueous oregano extract disclosed flavonoids and tannins [31], in alignment with our study. These compounds, reported for their antioxidant properties, have been documented in both aqueous and ethanolic extracts, as well as in oregano essential oil, further amplifying the biosensing potential of the present study’s findings [32,33,34,35]. A recent study has already demonstrated that it is also possible to evaluate and quantify these compounds with antioxidant activities through a biosensing approach [36]. Lavender extract, recognized for its cytotoxicity, demonstrated the presence of tannins and saponins [37], a finding consistent with our present study, along with the discovery of flavonoids. Notably, wormwood and sage extracts exhibited saponins exclusively, which have been harnessed within liposomes to activate a cancer response system [38].

The assembly of nanometric structures was observed across all formulations, encompassing either empty or full liposomes of all evaluated plant extracts. Lavender species exhibited a minimal reduction in size when transitioning from empty to full liposomes. In contrast, wormwood and oregano species, when empty, displayed bimodal subpopulations, which transitioned to a single nanometric population when full. Notably, sage species exhibited statistically significant growth in liposome size when full, underscoring the intricate dynamics involved. Some pieces of evidence have indicated that the predicted maximum EE does not consistently result in a substantial alteration in liposome size [23]. This observation aligns with the outcomes of the current study, where this phenomenon was observed in liposomes loaded with lavender, wormwood, and oregano extracts, although not in the case of sage extract.

When comparing the salvia full liposome to the extract alone, distinct differences were evident in terms of HD and PdI. However, the ZP and Cnd-EM remained statistically unchanged, once again highlighting the insufficient EE for the extract of this particular species. This trend resonated with research involving other nanostructured entrapment systems, wherein a low EE did not yield notable alterations in ZP [39]. The observed increase in PdI and HD could be attributed to the presence of phospholipids within the sample, which hindered the formation of concentric vesicles necessary for the expected encapsulation process.

Upon evaluating the ZP, as an increase in the ZP modulus can indicate interactions between active agents and phospholipids, thereby indicating encapsulation success [40], we observed distinctive changes in the liposome samples containing lavender, wormwood, and oregano extracts. These statistically significant increases in the ZP modulus, when compared to the extract, suggested the potential encapsulation of bioactive compounds. This finding was further supported by the EE results. Additionally, this trend was not confirmed for sage, for which the change in the ZP modulus was not statistically significant.

Previous studies have revealed the correlation between Cnd and particle properties [41,42,43]. However, a specific Cnd parameter, relevant to real-world applications of EE as demonstrated in this study, had not yet been explored. A substantial increase in Cnd-EM was observed in full liposomes compared to the extract, specifically in samples with higher encapsulation rates, such as wormwood and oregano. These findings corroborated earlier research [43], further reinforcing the biosensing-driven connection between Cnd and EE. Notably, the lavender sample exhibited a decrease in Cnd-EM upon full liposome encapsulation compared to the extract, while no statistically significant change in Cnd-EM was observed in the sage sample. This also underscored the correlation between Cnd-EM and EE in this study. Additionally, a study has reported that an increase in particle size may result in an unchanged or decreased Cnd [43], as observed in the sage sample. Furthermore, evaluations have demonstrated that the Cnd of extracts may not significantly vary over time [44].

Our findings provide compelling evidence of the effectiveness of the platinum conductivity electrode in establishing a linear response (r^2^ ≥ 0.950) correlated with the extract concentration in a solution. This accomplishment overcomes the challenge of quantifying EE in liposomes containing non-isolated contents, making a notable advancement towards biosensing applications.

## 5. Conclusions

This study highlighted the profound interplay between EE and Cnd, resulting in a robust correlation (r^2^ ≥ 0.950). This harmonious relationship allows the calculation of encapsulation in liposomes hosting intricate extracts, thereby reinforcing the biosensing paradigm. Moreover, the alignment of the ZP and HD with EE yielded profound insights, further solidifying the interface between biosensing and nanobiotechnology.

The implications of these findings will reverberate deeply within the realm of nanobiotechnology, ushering in an era of groundbreaking carrier nanosystems. The connection between EE and Cnd provides an outstanding toolkit to harness the potential of encapsulating complex extracts, transcending prior limits.

In essence, the precise determination of liposomal EE, even amidst the intricacies of non-isolated plant extracts, paves an uncharted path in drug delivery and the broader domain of biomedical applications. This newfound capability nurtures an environment ripe for innovative therapeutics and transformative biomedical interventions.

In conclusion, this study is a resounding testament to the importance of EE within the nanosystem landscape, intricately woven into the tapestry of Cnd. As we unravel the complexities of liposomes laden with diverse botanical extracts, a boundless realm of possibilities will unfold for extract delivery and disease treatment. This catalytic synergy between biosensing and nanobiotechnology charts a compelling trajectory, poised to orchestrate epochal advancements at the frontiers of scientific exploration.

## Figures and Tables

**Table 1:** Reactions carried out for the characterization of secondary metabolites.

Phytochemical Group	Reactions Performed
Flavonoids	Shinoda’s reaction
	Aluminum chloride reaction
	Ferric chloride precipitation
	Characterization of anthocyanins
Anthraquinones	Direct Borntrager reaction
	Indirect Borntrager reaction
	Ferric chloride precipitation
Tannins	Ferric chloride reaction
	Protein reaction
	Precipitation of alkaloids
	Reaction with copper acetate
	Reaction for condensed tannins
Saponins	Foam foaming
	Resistant foam formation

**Table 2:** Qualification of secondary metabolite classes presence or absence based on each reaction.

Phytochemical Group	Reactions Performed	Lavender	Wormwood	Oregano	Sage
Flavonoids	Shinoda’s reaction	+++	+	++	++++
	Aluminum chloride reaction	−	−	+	−
	Ferric chloride precipitation	++	+	+++	++
	Characterization of anthocyanins	+	−	++	−
Anthraquinones	Direct Borntrager reaction	−	−	+	−
	Indirect Borntrager reaction	−	−	−	−
	Ferric chloride precipitation	−	−	−	−
Tannins	Ferric chloride reaction	+++	++	+++	+++
	Protein reaction	++	−	++	−
	Precipitation of alkaloids	++	++	++	++
	Reaction with copper acetate	++++	++	++++	++++
	Reaction for condensed tannins	++	−	−	−
Saponins	Foam foaming	++	+	+	+++
	Resistant foam formation	+	+	−	++

Note: “+” indicates the presence of the secondary metabolite class; “++” indicates the presence of the secondary metabolite class visually more expressive than +; “+++” indicates the presence even more visually expressive than ++; “++++” indicates a stronger presence of the secondary metabolite class; “−” demonstrates that the reaction did not indicate the presence of the metabolite. A greater number of “+” signs indicates a more visually expressive reaction.

**Table 3:** Polydispersity index (PdI), zeta potential (ZP), and electrical conductivity (Cnd-EM) for the extract, empty liposome, and full liposome. The values represent the arithmetic mean and standard deviation of the mean values obtained from three independent readings.

	Sample	Lavender	Wormwood	Oregano	Sage
PdI	Extract	0.385 ± 0.004	0.406 ± 0.007	ø	0.657 ± 0.069
	Empty liposome	0.604 ± 0.017 ^ac^	0.460 ± 0.027	0.686 ± 0.104	0.318 ± 0.034 ^ac^
	Full liposome	0.407 ± 0.044	0.444 ± 0.047	0.488 ± 0.056 ^b^	0.984 ± 0.028 ^ab^
ZP (mV)	Extract	−14.0 ± 0.6	−23.3 ± 0.5	−2.7 ± 0.4 ^bc^	−18.9 ± 1.6
	Empty liposome	−14.4 ± 0.6	−22.2 ± 2.2	−28.2 ± 0.8	−11.5 ± 1.5 ^ac^
	Full liposome	−34.4 ± 3.3 ^ab^	−32.3 ± 4.0 ^ab^	−27.1 ± 4.8	−21.1 ± 0.5
Cnd-EM (ms/cm)	Extract	0.288 ± 0.004 ^bc^	0.220 ± 0.002 ^bc^	0.299 ± 0.005 ^bc^	1.410 ± 0.077 ^bc^
	Empty liposome	0.006 ± 0.001 ^ac^	0.009 ± 0.001 ^ac^	0.015 ± 0.063 ^ac^	0.014 ± 0.005 ^ac^
	Full liposome	0.230 ± 0.003 ^ab^	0.238 ± 0.002 ^ab^	0.322 ± 0.005 ^ab^	1.420 ± 0.077 ^ab^

Statistical analysis was conducted using paired comparisons by one-way ANOVA (*p* < 0.05). Comparisons were made among samples of the same species, including: empty liposomes (containing only water), filled liposomes (containing aqueous plant extract), and the original extract. ^a^ indicates a sample that was statistically different from the extract of its own species; ^b^ indicates a sample that was statistically different from the empty liposome of its own species; ^c^ indicates a sample that was statistically different from the full liposome of its own species.

**Table 4:** Enhanced biosensing analysis of electrical conductivity in ultra-filtered extracts and liposomes. Comparison between the expected electrical conductivity if the liposome had 100% unloaded extract and ultrafiltered extracts from full liposomes.

Sample	Electrical Conductivity if Extract 100% Unloaded (µs/cm)	Electrical Conductivity in Ultrafiltered Liposomes (µs/cm)
Lavender	3499.55 ± 14.31	1558.25 ± 13.21
Wormwood	21,609.75 ± 243.25	1688.52 ± 32.83
Oregano	18,822.80 ± 29.14	2113.97 ± 38.48
Sage	9222.80 ± 28.67	8554.18 ± 139.85

Arithmetic mean and standard deviation of the mean values acquired from three manual electrical conductivity readings. Values were adjusted by subtracting both water and empty liposome for ultrafiltrate values.

## Data Availability

Not applicable.
